# A Parent-Based Intervention for Reducing High-risk Social Media Cognitions, Alcohol Use, and Negative Consequences Among Adolescents: Protocol for a Randomized Controlled Pilot Study

**DOI:** 10.2196/38543

**Published:** 2022-05-17

**Authors:** Dana M Litt, Femke Geusens, Abby Seamster, Melissa A Lewis

**Affiliations:** 1 School of Public Health University of North Texas Health Science Center Fort Worth, TX United States; 2 Leuven School for Mass Communication Research KU Leuven Leuven Belgium; 3 Research Foundation Flanders (FWO Vlaanderen) Brussels Belgium

**Keywords:** parent-based interventions, alcohol, pilot study, social media, mobile phone

## Abstract

**Background:**

The prevalence of adolescent alcohol use continues to be a public health concern. Although adolescents spend an increasing amount of time with their friends, parents remain an important source of support and continue to play a key role in the lives of their adolescents. Extensive research in this area has resulted in parent-based intervention (PBI) efforts to prevent or reduce adolescent alcohol use. However, one major limitation of PBIs is that they do not currently consider the large role that social media plays in adolescents’ lives and in relation to their alcohol use. We will add to the literature by developing and refining a web-based PBI designed to reduce both high-risk social media cognitions and alcohol use among adolescents.

**Objective:**

The central goal of the proposed study is to develop, refine, and pilot a web-based PBI to reduce both high-risk social media cognitions and alcohol use among adolescents.

**Methods:**

A total of 100 parent-teen dyads will be randomly assigned to one of the following 2 conditions: intervention or control. Parents in the intervention group will be given access to the web-based PBI and suggestions for working through the PBI modules with their teens. The parent-teen dyads will fill out 3 questionnaires: a baseline questionnaire, 1-month questionnaire, and 6-month questionnaire.

**Results:**

Recruitment and enrollment will begin in August 2022. Upon completion of the intervention trial, we will examine the feasibility, acceptability, and preliminary effect sizes of the newly developed web-based PBI.

**Conclusions:**

This study has the potential to open doors for future studies examining the clinical implications of an efficacious web-based PBI to reduce alcohol use and high-risk cognitions about alcohol displays on social media.

**Trial Registration:**

ClinicalTrials.gov NCT04333966; https://clinicaltrials.gov/ct2/show/NCT04333966

**International Registered Report Identifier (IRRID):**

PRR1-10.2196/38543

## Introduction

### Background

The prevalence of adolescent alcohol use continues to be a public health concern [1]. Alcohol-related problems occur in school and interpersonal, social, or health domains [2]. Almost 25% of 14- to 15-year-olds drink alcohol, with ≥90% of all alcohol consumed by adolescents being consumed in the form of heavy episodic drinking [3]. Research indicates that by 15 years of age, approximately 35% of teens have had at least one drink, and by 18 years of age, that number rises to 65% [4]. Past-year use of alcohol is reported by 17.6%, 38.3%, and 55.6% of 8th, 10th, and 12th graders, respectively [5]. Considering that alcohol is directly linked to the leading causes of death in adolescence, the US Department of Health and Human Services has listed the reduction of underage alcohol use as one of their major objectives of Healthy People 2030 [6]. This study protocol directly answers this call.

Although adolescents spend an increasing amount of time with their friends [7,8], parents remain an important source of support and continue to play a key role in the lives of their adolescents [9,10]. Extensive research in this area has resulted in parent-based intervention (PBI) efforts to prevent or reduce adolescent alcohol use [11]. Research has shown that teens whose parents received a PBI reported less alcohol use and fewer alcohol-related consequences up to a 9-month follow-up than controls [12-15]. However, one major limitation of PBIs is that they do not currently consider the large role that social media plays in adolescents’ lives and in relation to their alcohol use.

Most (90%) adolescents are on social media [16], and their Facebook, Instagram, and Twitter profiles include alcohol content [17,18]. Thus, adolescents are exposed to social media alcohol displays, which are associated with high-risk cognitions and alcohol use [19-24]. Research has argued that existing parental mediation techniques grounded primarily on television and film media have fundamental inadequacies when applied to media such as websites, social media, and mobile apps, as they do not account for the interactivity, immersive web-based environments, and mediated communication innate to social media [25]. Furthermore, most PBIs are presented in a static manual form [15,26,27]. We are unaware of any study to date that has developed and tested a PBI about alcohol use and the role of social media in adolescent alcohol use. As such, we will add to the literature by developing and refining a web-based PBI designed to reduce both high-risk social media cognitions and alcohol use among adolescents.

### Adolescent Alcohol Use

Research concerning the initiation and progression of adolescent alcohol use indicates that most youth initiate use by experimenting with alcohol during early adolescence and that such early experimentation can lead to later heavy alcohol use [28-33]. Early initiation of alcohol use is also associated with various additional negative outcomes in later adolescence and early adulthood such as violent and delinquent behavior, poor physical health, and mental health problems [31,32]. As this general pattern of alcohol use initiation and escalation is well documented, many prevention programs for adolescent alcohol use aim to prevent early-stage alcohol use or at least delay the initiation or onset of alcohol use among adolescents. Given how prevalent and potentially harmful adolescent alcohol use is, and the fact that studies have not indicated increased alcohol involvement following universal interventions [34,35], universal prevention of alcohol use is an appropriate choice for this age group [36].

Preventing underage alcohol use and reducing the proportion of people who engage in heavy drinking are listed as major objectives of Healthy People 2030 [6]. To address these issues, it is important to consider factors that may be related to initiation and progression of alcohol use among adolescents. Parent-teen communication familial factors have frequently been focused on in relation to adolescent development and have been shown to inform effective interventions [37]. Although adolescents usually strive to become more autonomous from their parents and spend an increasing amount of time with their friends [7,8], parents remain an important source of support and continue to play a key role in the lives of their adolescents [9,10].

A growing body of literature provides evidence for reductions in adolescent drinking associated with parental influences, including greater parental monitoring [38-40], less favorable parental attitudes and beliefs about drinking [41,42], and more positive quality of the parent-child relationship and communication [43-45]. Miller-Day and Kam [46] found that targeted parent-teen communication, defined as “direct and indirect, as well as one-time and ongoing, conversations specifically about alcohol,” was associated with lower levels of adolescent drinking. In addition, Wood et al [47,48] discovered that parental communication variables were related to lowered alcohol use and related problems. These findings illustrate that parental influence is relevant to adolescent decision-making regarding alcohol use, even as their children transition from adolescence to young adulthood. As parents are likely to exert a major influence on their teens’ behavior including their substance use, parents are important agents who should be included in intervention efforts.

### Social Media and Adolescent Alcohol Use

Aided by the convenience and constant access provided by mobile devices, especially smartphones, research indicates that 89% of teens report going on the internet at least multiple times per day, including 45% who say they go on the internet *almost constantly*, and much of this time is spent on social media [49]. Adolescents are the age group that uses social media the most, with research showing that up to 97% of people in this age group are members of at least one social media platform. Although Facebook used to be the most commonly used social media platform among adolescent users, only a few years ago, it has rapidly declined in popularity (it is currently used by 51% of adolescents). Currently, the top 3 platforms are YouTube (used by 85%), Instagram (used by 72%), and Snapchat (used by 69%) [49]. Notably, almost 5 times as many adolescents use social media (29%) instead of email (6%) for daily communication [16], indicating that social media is a central way in which adolescents communicate with peers.

A significant number (between 20% and 30%) of adolescent social media profiles include alcohol-related content or displays, with most displays being proalcohol or favorable toward heavy alcohol use [17,18,20,23]. This indicates that adolescents are both the creators and viewers of alcohol-related content on social media platforms. Alcohol-related displays on social media have repeatedly been found to be associated with adolescent problem drinking [21,22,24,50-52]. There is a robust relationship between exposure to social media alcohol content and alcohol consumption 6 months later, which persists even after close friends’ drinking is accounted for [53]. These findings indicate that alcohol references on social media do not simply reflect alcohol use behaviors that would otherwise be observed in the absence of social media.

Given how much time adolescents spend on social media in conjunction with the multiple ways (eg, messaging and photos) and opportunities to communicate about alcohol on social media, social media are likely to be important social influences related to alcohol use [54-56]. Research has shown that social media contributes to the salience and amplification of drinking events, as people are now exposed to new and different drinking groups and locations than they would be offline [57]. In fact, research has shown that adolescents report having more social media contacts than their offline peers [58], which expands exposure to peer risk behavior, including alcohol use. In addition, research has shown that an increase in the number of Facebook friends is significantly associated with an increase in one’s own alcohol displays [59]. This becomes particularly important because both experimental and longitudinal research indicates that viewing social media profiles that contain alcohol displays is related to increased risky drinking cognitions [19] and high-risk alcohol use [60,61]. In essence, exposure to alcohol content posted by friends can cultivate unfavorable cognitions such as norms, perceived vulnerability to risk, and attitudes that are then rapidly spread through the web-based networks and contribute to the adoption of risky beliefs and behaviors among other adolescents. Several studies suggest that adolescents use social media to reconstruct negative and risky drinking practices into positive outcomes [54,62] to avoid acknowledging any implications of or reference to negative consequences associated with drinking [63]. Furthermore, research suggests that adolescent viewers are likely to accept their peers’ social media posts as accurate representations of their offline experiences [64].

Research has shown that alcohol use and alcohol-related displays on social media are common among adolescents and that these displays are associated with problematic drinking; therefore, alcohol intervention research for this age group should include content directly related to these social media influences on alcohol use. Previous research has shown that parents have limited insight into the types of experience teens have on the internet [65,66]. Although research indicates that many parents try to monitor social media activities [67], a study has documented the limited efficacy of such attempts [68], concluding that the most effective way for parents to gain insight into their teens’ web-based activity is for their teens to tell them [69]. This effort to explain the role of parents as socialization agents in teens’ media use is referred to as parental mediation [70].

Two broad dimensions of parental mediation have been examined: restrictive mediation and instructive mediation [71]. Restrictive mediation refers to parents limiting their children’s access to media or setting rules in terms of appropriate media content and the amount of media exposure permitted [72]. Instructive mediation refers to parents explaining and discussing undesirable aspects of media consumption, suggesting proper ways to use media, and overall communication meant to help their teens understand the nature and possible impact of media messages [70,73,74]. The parental mediation literature suggests that instructive mediation is more effective than restrictive mediation in reducing undesirable influences of social media on teens (refer to the study by Valkenburg et al [74]), partly because instructive mediation is based on conversation and critical discussion between parents and teens, which is more likely than control-based restrictive mediation to cultivate critical thinking skills and skepticism in teens [73].

In general, the observational nature of traditional media channels (ie, television, film, and print), meant that audiences were passive observers who were not able to influence the content they were observing [75]. However, social media has created a culture in which users can participate in content creation and sharing. As social media are more interactive and repetitive than other types of media (eg, television, film, and print), contain images of actual peers, and content can both be created and consumed, the risks related to social media may be greater than those related to other forms of media [19]. Although many parents may feel comfortable with the social media that their children are using, others may find it difficult to relate to their digitally savvy children for several reasons. First, parents may lack a basic understanding of social media socialization [76]. In addition, most parents may not understand that for many adolescents, their web-based lives are an extension of their offline lives, and as a result, there may be a disconnect between parents and their teens in the understanding of social media use [77]. As such, strategies used for general parental mediation of more traditional forms of media (ie, television, film, and print) may not be adequate to account for the unique risks presented by social media. In fact, research has argued that existing parental mediation techniques grounded primarily on television and film media have fundamental inadequacies when applied to media such as websites, social media, and mobile apps, as they do not account for the immersive web-based environments and mediated communication innate to social media [25]. Furthermore, most parental mediation studies involving social media focus on issues such as privacy [71,78] and cyberbullying [79] and do not take into account the role that social media plays in relation to underage alcohol use.

Only 2 studies have provided an alcohol intervention via social media [80,81], but they did not address any content related to social media. Rather, these studies used social media as a mode of intervention delivery. A systematic review that focused on the use and effectiveness of social media in health behavior change found that Facebook is the most commonly targeted social media in this type of intervention [82], despite teens being on many other social media. Weight loss and eating behaviors were the most commonly targeted health behaviors, with only a few studies targeting health risk behaviors (smoking and condom use). None of the studies identified in the review targeted alcohol use, indicating a substantial need for this research. We are unaware of any study that has tried using parental intervention as a strategy to reduce alcohol use and alcohol displays on social media.

### Development of a Web-Based PBI Focusing on the Role of Social Media in Adolescent Alcohol Use

PBIs are universal prevention programs that target adolescent behaviors but use the parents of adolescents as change agents [14]. This is done by advising parents on how to engage in instructive parental mediation to become effective change agents and to help parents implement best practices in communication. For example, parents sometimes know less than their children do about new technologies or popular topics [83], and parents may be uncomfortable or unwilling to discuss content that may lead to conflict. Therefore, the goal of PBIs for alcohol use is to encourage parental mediation by educating parents about the current landscape related to alcohol, issues their teens may be facing, and advising parents on how to initiate and conduct healthy communication.

At the core of PBI research is the assertion that parents are a major source of health information for their teens and that the majority of teens report being very satisfied with the information they receive from their parents [84]. Moreover, providing parents with information to share with their teens will allow teens to discuss and clarify, which would not occur if teens were provided the information directly. According to national agency, Mothers Against Drunk Driving, in 2017, a total of 9100 parents attended in-person workshops (Mothers Against Drunk Driving, email, April 10, 2018). This indicates that despite their busy schedules and competing tasks, parents are interested in receiving more information about how to talk to their teens about substance use. Therefore, a PBI that includes information about alcohol and social media is likely to be an important source for parents and their teens to receive and share information related to social media.

In a typical PBI, handbooks on how to communicate with adolescents about alcohol are mailed to the parents of adolescents (most commonly incoming college freshmen) [12,14,27,85]. Specifically, the theory underlying PBIs emphasizes 2 key components: style and content. Parents are instructed to use an empathic and conversational communication style while providing accurate information about student alcohol consumption (eg, biological aspects). Components generally included in PBIs involve those based on the theory of planned behavior (eg, attitudes, norms, and perceived behavioral control) and those based on facilitating healthy communication and relationships between a teen and their parent [14]. A major strength of PBIs is that parents can tailor the content and timing of communication to adolescents based on their knowledge of their child’s strengths, weaknesses, maturity level, and current alcohol use. This feature of PBIs respects the diversity of adolescents and their experiences and recognizes that not all adolescents respond identically to the same information. A PBI, as opposed to formal workshops, provides parents with more time flexibility and permits them to work at their own pace and during times that they choose.

This approach has proven successful in reducing the odds that nondrinking high school students will initiate alcohol use during the first year of college [12] as well as reducing general alcohol consumption [12,14,15,27,86]. Although these effects may be modest, given the ease of dissemination, low cost, and complex nature of underage drinking, PBIs are supported as a model resource in the National Institute on Alcohol Abuse and Alcoholism’s College Intervention Matrix and by the Surgeon General [2]. A systematic review [26] supported the idea of involving parents in prevention programs. Across studies and concepts, they found evidence that participating in PBIs had desirable effects on parenting measures such as rule-setting, monitoring, and parent-child communication as well as the prevention and reduction of adolescent substance use.

Despite promising findings for PBIs to reduce adolescent alcohol use, one limitation of this body of work is that they do not consider the increasing role that social media plays in the lives of adolescents and how this is associated with their alcohol use. In addition, most PBIs consist of static formats such as downloadable or mailed parent handbooks [15,26,27] as opposed to web-based formats, and therefore, they do not provide continued support throughout the PBI. Furthermore, the nature of social media lends itself to creating PBI materials that can give parents a realistic view of the web-based environments that their children are navigating. We know that there is a strong relationship between alcohol use and viewing and creating alcohol displays on social media and that parents are still an important channel of communication about alcohol use and social media for adolescents. The next logical step is to determine whether PBIs, and in particular a PBI containing both web-based content and SMS text message prompts, can be developed to reduce both alcohol use and risky cognitions related to alcohol displays on social media. This is the main goal of this intervention protocol.

## Methods

### Study Design

This study will involve conducting a pilot study with 100 parent-teen dyads to determine the feasibility, acceptability, and preliminary effect sizes (to estimate power and sample sizes for a future full-scale randomized controlled trial [RCT]) of the developed social media PBI. A total of 100 parent-teen dyads will be randomized into 2 groups: a group receiving the newly developed web-based social media PBI or an active control group (treatment as usual [TAU] [2]). We hypothesize that the social media PBI, relative to TAU, will be rated by parents and teens as more *feasible* (number of eligible participants, number of parents who gave consent, number of teens who gave consent, time taken to achieve planned recruitment and enrollment goal, and rate of study attrition) and more *acceptable* (measured at 1-month follow-up with specific postintervention survey items, ie, proportion of parents and teens who find the intervention acceptable; ease of interacting with social media PBI content; relevance of material; finding content helpful, beneficial, and important; ratings of individual modules and components of the social media PBI; the proportion of parents and teens who would recommend the study to other families; and the proportion of parents and teens who found the social media PBI to be favorable overall) in relation to active control or TAU. We further hypothesize that teens and parents in the social media PBI condition will report more positive communication about alcohol and social media at 1- and 6-month follow-up relative to TAU.

*Parent hypotheses*: We hypothesize that at 1- and 6-month follow-up, parents in the social media PBI condition will report greater knowledge about alcohol as well as the role of social media in alcohol use relative to TAU.

*Teen hypotheses*: We hypothesize that teens in the social media PBI condition will report less drinking, fewer alcohol-related negative consequences, less favorable attitudes toward posting about alcohol on social media, greater perceived vulnerability to the risks of posting alcohol displays on social media, and decreased normative perceptions about how many teens post alcohol displays on social media relative to TAU at 1- and 6-month follow-up.

### Ethics Approval

This study was reviewed and approved by the North Texas Regional Institutional Review Board (#2021-124). All participants will sign an approved consent form in accordance with the ethical standards of Helsinki.

### Recruitment

**Figure 1:**
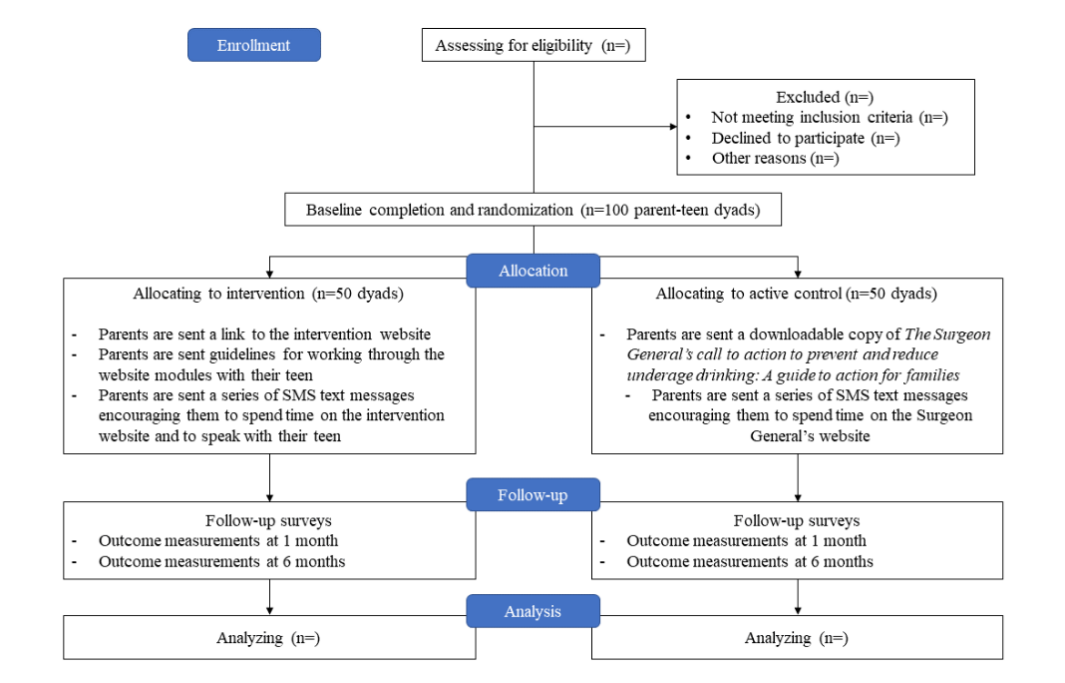
Pilot study workflow diagram.

We will use a multimethod approach to reach a wide cross section of parents of adolescents and young adults from Texas, including web-based and electronic newspaper advertisements, electronic flyers, and social media. Web-based advertisements will be placed in local and social media outlets frequented by those likely to have children aged 15 to 20 years. We will also recruit parents through community organizations in major cities in Texas. Contacts at community organizations will be initially contacted via email by project staff. In this email, the project staff will provide information about the study and encourage further questions. If they agree to share study information with their members, the project staff will send them the consent link and QR code. Similar emails will be sent to high school administrators to recruit the parents of students. We will stratify recruitment based on age, gender, and ethnicity, recruiting equal number of parents with adolescents in each age category (ie, 15, 16, 17, 18, 19, and 20 years) and targeting equal number of males and females in each age group. We will recruit all minority parents to be above the local census estimates. Figure 1 shows the workflow diagram.

After receiving information about the study, participants will be presented with the web-based informed consent statement that covers the screening survey, intervention, and follow-up surveys. If eligible, individuals will be asked to provide an electronic signature before being directed to participate in the web-based screening survey, which will determine whether they are a good fit for the study (refer to Textbox 1 for the inclusion criteria). Only those participants who sign both the web-based consent and assent form and Health Insurance Portability and Accountability Act authorization form (one for themselves and one for their child if the child is aged 15-17 years) will be routed to the Screening Welcome Page. Parents are informed in the parent consent form that their teen will be asked to sign their own consent and assent form to indicate whether they want to participate in the research study. Once a parent has been deemed eligible and has provided contact information (and consent and Health Insurance Portability and Accountability Act form for their teen aged 15-17 years), their teen will be sent a link (using the contact information provided by their parent) to the consent form and screening survey. As with the parents, adolescents will be presented with an web-based informed consent statement and will be asked to provide an electronic signature before being directed to participate in the web-based screening survey, which will determine whether they are a good fit for the study (Textbox 1).

We will use previously successful strategies to maximize retention [87-91]. First, study incentives will show that time and effort are appreciated. We pay for all assessments (with bonuses) to encourage high completion and send all participants a variety of nominal items intended to offset the cost of completing assessments, thus promoting participation. Second, data collection strategies allow participants to complete surveys using smartphones or computers. Third, we will call participants before data collection sessions (eg, 1- and 6-month follow-up surveys) as a reminder of an upcoming survey and to stay connected with the project staff. Fourth, we will gather contact information at each time point and provide a toll-free number, study email address, and study website with a link for participants to update the contact information. Fifth, we will use persistent monitoring to keep track of barriers to participation. These methods have proven effective in our ongoing successful study, retaining adolescent and young adult participants for over 2 years.

**Inclusion criteria.**

**Inclusion criteria for parents or legal guardians**

- Have a child aged between 15 and 20 years who currently lives with them
- Believe that their child is active on at least one social media platform
- Live in Texas
- Provide a valid email address
- Own a cellphone with SMS text messaging capabilities and be okay with receiving messages
- Provide consent for their child if the child is aged 15 to 17 years
- Provide valid contact information for their child
- Be willing to participate in a study that involves a parenting program and a series of web-based surveys with their teen
- If multiple parents are interested, they will be instructed to choose one parent to participate

**Inclusion criteria for teens**

- Have an eligible parent with whom they currently live
- Be aged between 15 and 20 years
- Live in Texas
- Be active on at least one social media platform
- Provide a valid email address
- Own a cell phone with SMS text messaging capabilities and be okay with receiving messages
- Be willing to complete three 45-minute web-based surveys over the course of 6 months

### Procedures

**Figure 2:**
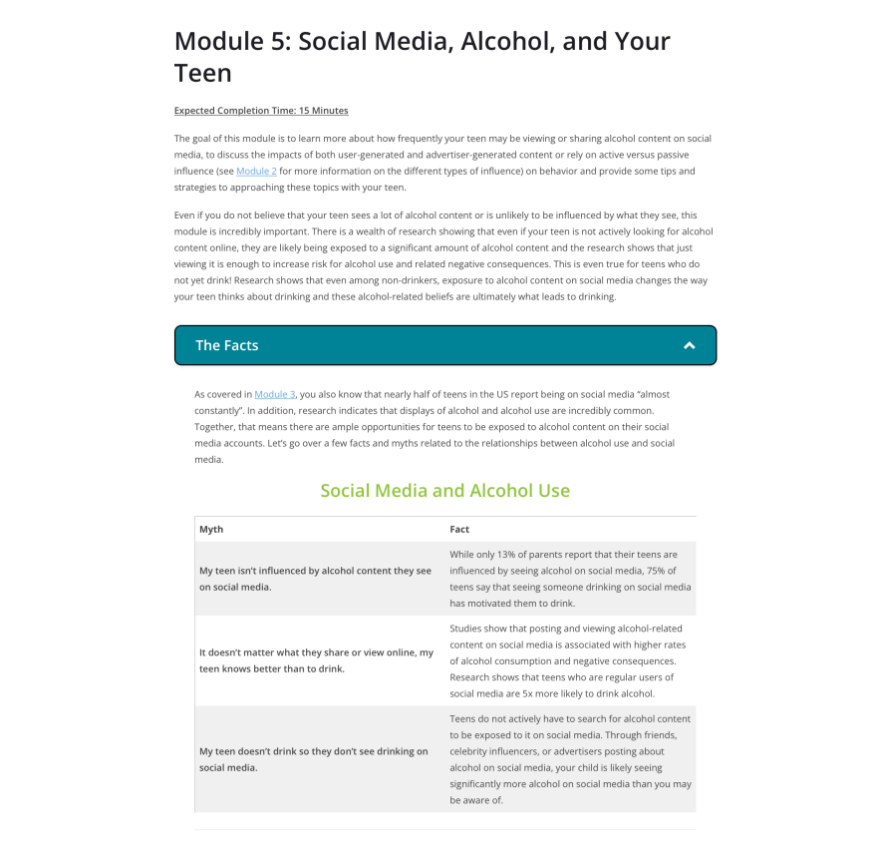
Sample module content.

After both the parent and teen in each dyad have completed their respective baseline surveys, the parent-teen dyads will be randomly assigned to one of the following 2 conditions: PBI or control. Parents in the intervention condition will be sent an email and a SMS text message containing a link to the PBI website along with information explaining the study and providing guidelines for working through the modules with their teens (refer to Textbox 2 for an overview of the specific modules; see Figure 2 for sample module content). Parents may revisit the web-based PBI as many times as they like over the course of a month before the 1-month survey. Parents will also be sent a series (4 total) of SMS text messages encouraging them to spend time on the web-based PBI and encouraging them to speak with their children. Parents in the control condition will be sent an email with a downloadable copy of *The Surgeon General’s Call to Action to Prevent and Reduce Underage Drinking: A Guide to Action for Families*. This manual is publicly available on the Surgeon General’s website [2]. Parents in the control condition will be sent a series of SMS text messages (4 total) encouraging them to spend time reviewing the Surgeon General’s guide. Parents in the control condition will be provided the link to the intervention condition website at the end of study completion.

Parent-teen dyads who meet the inclusion criteria and pass phone verification (to confirm identity) will be emailed and texted a baseline survey link. The baseline survey will include questions about demographics, social media literacy, parenting, drinking and drug use, drinking cognitions, mental health, and other health behaviors and will take approximately 45 minutes to complete. The questions in the assessments will also include topics about sexual orientation, gender identity, religion, and relationship status. For those in the intervention group, items in the 1-month follow-up survey will assess satisfaction with the intervention website. If we do not receive the completed assessment at each follow-up (baseline assessment, 1-month assessment, and 6-month assessment), we will periodically send reminders—via email (up to 6), SMS text messages (up to 6), and phone or voicemail (up to 5). Parents and teens can earn US $25 for the baseline assessment, US $35 for the 1-month assessment, and US $40 for the 6-month assessment, meaning that all participants can earn up to US $100.

**Parent-based intervention’s modules and descriptions.**

**Module 1: communication matters**

- Parents will learn about facts and myths related to communicating with their teen as well as review tips and strategies to effectively initiate and maintain conversations.

**Module 2: your teen’s world**

- Parents will gain insight into the important role of social influence on teen decision-making.

**Module 3: talking about social media with your teen**

- Parents will learn about specific tips and strategies related to talking with their teens about their social media use.

**Module 4: media literacy for teens**

- Parents will review the importance of teaching their teens social media literacy skills and gain tips and strategies on teaching and practicing these skills.

**Module 5: social media, alcohol, and your teens**

- Parents will be exposed to information related to the impact of alcohol content their teen may view or share on social media on their behavior and learn tips and strategies for approaching these topics with their teen.

**Module 6: social media and other health risk behaviors**

- This module will review other concerns (drug use, sexual behavior, mental health, and cyberbullying) that parents may also want to discuss with their teens.

**Module 7: closing thoughts**

- A summary of topics covered and final tips and strategies will be provided.

**Module 8: resources**

- Additional resources related to the topics discussed across all modules are available here.

### Measures

Behavior will be reported over lifetime (baseline) and the past month (1- and 6-month follow-up) to reduce problems with retrospective recall and overlap. All measures will be assessed at baseline and 1- and 6-month follow-up, unless otherwise noted.

### Demographics

Demographics will include age, biological sex, gender, race, ethnicity, height, weight, and family history characteristics.

### Parent-Teen Relationships

Parent-teen relationships will be evaluated in terms of parent-teen communication regarding both alcohol and social media (Cronbach *α*=.53-.75) [10]. To determine the nature of parental involvement and monitoring of teens, parental monitoring will be assessed using the Parental Monitoring and Knowledge Scale (Cronbach *α*=.81) [92] and the Parental Monitoring of Social Media measures (Cronbach *α*=.67-.88) [93].

### Social Media Use

Both parents and teens will be asked about their own social media use by answering questions on how often they check different social media platforms, their exposure to alcohol-related social media content, their own alcohol-related communication on social media, and their motives for using social media. Parents will also report perceptions of their teen’s social media use with the same questions.

### Alcohol and Other Substance Use

Parents and teens will be asked questions regarding their alcohol and substance use. Family history of alcohol (baseline only) as well as lifetime and past-year alcohol use (baseline only) will be assessed [94]. Drinking will be assessed using the Daily Drinking Questionnaire and the Quantity Frequency Index (Cronbach *α*=.73) [95-97] and the Alcohol Use Disorders Identification Test (Cronbach *α*=.85) [98,99]. Alcohol consequences will be assessed using the Young Adult Alcohol Consequences Questionnaire (Cronbach *α*=.79) [100]. Marijuana use will be measured using items including lifetime, past-year, and past-month marijuana use [94]. Other substance use will be assessed for lifetime and past-month use with the Customary Drinking and Drug Use Record (Cronbach *α*=.70-.94) [101].

### Satisfaction With the Intervention

Finally, both parents and teens in the intervention group will be asked to complete a 1-month satisfaction questionnaire related to their experience, perceptions, and interactions with the intervention website. In particular, parents will be asked how often they visited the web-based PBI and its specific modules; how they feel about the PBI and the modules in terms of acceptability (eg, acceptability of content delivery method), usability (eg, ease of viewing and interacting with PBI content), relevance (eg, relevance of material), and helpfulness (eg, finding content helpful, beneficial, and important); and whether parents would share the information in the PBI with anyone else. Both parents and teens will be asked whether they would like to have additional conversations on this topic, if they would recommend the study to others, and whether they found the program to be favorable overall.

### Statistical Analyses

To evaluate the pilot study proposed in this protocol, we will examine recruitment and retention rates, parents’ postintervention feedback as measured at 1-month follow-up (ie, accessible, usable, convenient, relevant, and helpful), teens’ rates of alcohol initiation and use and alcohol-related negative consequences, and parents’ and teens’ reports of alcohol and social media–related communication that will provide base rates and variance in outcomes to determine adequate power for a future full-scale efficacy RCT.

*Feasibility* will be assessed by the proportion of parents who meet the inclusion criteria and enroll for the study, the proportion of teens who meet the inclusion criteria and enroll for the study, and the proportion of parents and teens who complete the social media PBI at the 1-month follow-up. Finally, the time taken to recruit our target enrollment number will also be used as an outcome of feasibility.

*Acceptability* will be assessed with parents’ and teens’ responses at 1 month. Acceptability will be determined by (1) the proportion of eligible parent-teen dyads enrolled, with 80% (100/125) of eligible dyads agreeing to participate; (2) the proportion of participants (both parents and teens) who find the intervention acceptable (eg, acceptability of content delivery method), usable (eg, ease of viewing and interacting with PBI content), relevant (eg, relevance of material), and helpful (eg, finding content helpful, beneficial, and important); (3) parents’ and teens’ ratings of individual modules in the social media PBI; (4) whether parents and teens would like to have additional conversations on this topic; (5) whether parents would share the information in the PBI with anyone else; (6) the proportion of parents and teens who would recommend the study; and (7) the proportion of parents and teens who found the program to be favorable overall. Acceptability will be specifically determined if acceptability for the social media PBI is higher than that for the control and if at least 80% of responses in each domain are rated a 4 or higher (out of 5).

This pilot will *explore treatment differences and determine preliminary effect sizes* for teens’ drinking as well as parents’ knowledge about alcohol and social media, and parent- and teen-reported outcomes will be analyzed in separate models. All models will have 3 repeated measures (ie, baseline and 1- and 6-month follow-up), yielding up to 300 level 1 observations (repeated measures) across 100 level 2 cases (teens or parents). Before inferential statistics, univariate and bivariate descriptive statistics will be used to assess the distributions and simple associations among the variables. Primary teen-reported outcomes are alcohol use and negative consequences (both count outcomes) as well as cognitions (attitudes, norms, and perceived vulnerability related to social media alcohol displays; all modeled as normally distributed outcomes). Primary parent-reported outcomes will be knowledge about alcohol and social media (modeled as normally distributed outcomes). Given the repeated-measures design, generalized linear mixed models [102] will be used. Generalized linear mixed models (ie, hierarchical generalized linear models) allow for nonnormal outcomes and missing data.

## Results

This research was funded in August 2019, and the pilot RCT phase was approved by the institutional review board in January 2022. Recruitment and enrollment will begin in August 2022. The findings will be published in peer-reviewed journals and presented at international, national, or regional academic and professional meetings and conferences. This study is expected to conclude in August 2023.

## Discussion

### Principal Findings

The central goal of the proposed study is to develop, refine, and pilot a web-based PBI to reduce both high-risk social media cognitions and alcohol use among adolescents. Despite PBIs for alcohol use being widely accepted as efficacious, they do not take into account the growing importance of social media. Given how much time adolescents spend on the web and that their social media behavior is linked with alcohol use, PBIs could be more efficacious by addressing these social media sources of social influence. In preparation for large-scale prevention projects, pilot studies are needed to engage and solicit input from participant populations to empirically test and establish evidence for the feasibility and acceptability of intervention protocols. As such, this study will make important strides toward developing, refining, and establishing early-stage efficacy for a web-based social media PBI that can be tested in future randomized clinical trials.

This study has the potential to open doors to future studies examining the clinical implications of an efficacious PBI to reduce alcohol use and high-risk cognitions about alcohol displays on social media. An intervention designed to reduce an individual’s risky cognitions or beliefs based on alcohol displays that they view could, in turn, reduce risk cognitions among the individual’s web-based social media peer networks. Reducing these high-risk cognitions among one individual has the potential to reduce the number of displays their web-based peers view and thus potentially reduce peers’ drinking cognitions and ultimately behavior. This network-based cascade of peer influence can potentially reach thousands of adolescents from only a few initial participants. Determining an efficacious way to reduce high-risk alcohol display cognitions affords future research the opportunity to make use of social network-based interventions; thus, the proposed research has great potential to serve as a catalyst for future research.

Furthermore, because the social media PBI will be designed to display appropriately on any web-enabled device, including smartphones and tablets, there is strong potential to develop an app-based product (through commercial support or a subsequent grant) that could be implemented in different contexts, such as high school or college alcohol prevention, or as a stand-alone product. The knowledge gained from testing the feasibility, acceptability, and pilot of the developed social media PBI has a significant capacity to be generalized to interventions aimed at reducing other high-risk health behaviors (eg, marijuana use and other substance use) and could provide evidence that providing more PBI modalities, as opposed to being disseminated via emailed handbooks as is current practice, leads to greater reach, sustainability, and real-world impact. The prevalence of alcohol use in underage adolescents and young adults continues to be a public health concern [1]. People aged 12 to 20 years drink 11% of all alcohol consumed in the United States, with more than 90% of this alcohol being consumed in the form of heavy episodic drinking [103]. Excessive drinking is responsible for more than 4300 deaths among underage youth each year and cost the United States US $24 billion in economic costs in 2010 [104]. Thus, this study has a high potential to exert a sustained, powerful impact on the field of adolescent and young adult interventions for alcohol use, which remains prevalent and problematic.

In short, the proposed research has high potential for impacting the field of adolescent alcohol prevention, as it is both timely and innovative. With adolescents spending increasingly more time on social media and being exposed to more alcohol displays, and with research showing the association with increased drinking, it is imperative that interventions address the influence of social media. The proposed study is innovative, as it will be the first PBI to focus on the role of social media in adolescent alcohol use. Furthermore, because the proposed PBI will be designed to target alcohol displays as they pertain to multiple social media, not specific to any single social media, the PBI will be relevant to a wider group of adolescents as well as those who use more than one social media, and the intervention will continue to be relevant as social media come and go in popularity among adolescents. In addition, this study is particularly innovative, as it combines efficacious interventions based on parent-teen communication with the timely addition of alcohol displays on social media. Focusing the intervention on parent delivery means that the intervention can be available to adolescents and young adults in high school or following high school graduation. Under the National Institute on Alcohol Abuse and Alcoholism’s strategic plan, there is a request for research that expands screening and brief interventions to adolescent and young adult populations beyond that of 4-year college students [105]. Following high school, many young adults do not pursue postsecondary education or do not have access to brief interventions (as typically offered through university health centers). A PBI would allow delivery to parents of adolescents and young adults regardless of postsecondary attendance. Another particularly innovative aspect of this protocol is that unlike most PBIs that are sent to parents in a static manual format, the nature of the social media PBI (ie, the social media PBI being web-based with content and supplemented with SMS text messages) has the potential to make PBIs even more efficacious, as the parents will have continued support as they work through the social media PBI content with their teens.

### Limitations

No study is perfect; therefore, it is important to acknowledge potential limitations. First, a meta-analysis showed that the use of incentives to recruit for web-based research may lead to selection effects, which might impact external validity [106]. However, considering that random assignment ensures relatively similar characteristics across study conditions, selection effects are typically not a problem for randomized trials [107]. Second, all data will be collected in a single state, namely, Texas. It is likely that there exist different norms for adolescent alcohol consumption in different states and in different countries, which affect actual adolescent alcohol consumption and parental communication about alcohol consumption. Hence, the results may not be generalizable, and it is important to test this protocol in different settings when moving on to large-scale testing. Finally, considering that this is a small-scale pilot study, we did not design this study to be fully powered. Nevertheless, our results will provide preliminary effect sizes to calculate the power for a subsequent full-scale RCT.

### Conclusions

This study addresses the critical gap in the literature that PBIs do not take into account the large role that social media plays in the lives of adolescents and in relation to their alcohol use. Therefore, the main goal of our research is to determine whether a PBI can reduce both alcohol use and risky cognitions related to alcohol displays on social media. To achieve this goal, this research will collect pilot data to determine the feasibility, acceptability, and preliminary effect sizes of the developed social media PBI.

## Appendix

Multimedia Appendix 1 Peer-reviewer report from the Epidemiology, Prevention and Behavior Research Review Subcommittee - National Institute on Alcohol Abuse and Alcoholism (NIAAA) Initial Review Group (National Institutes of Health, USA).

## Abbreviations

NIAAA: National Institute on Alcohol Abuse and Alcoholism
PBI: parent-based intervention
RCT: randomized controlled trial
TAU: treatment as usual
