# Supplementary material for: A Parent-Based Intervention for Reducing High-risk Social Media Cognitions, Alcohol Use, and Negative Consequences Among Adolescents: Protocol for a Randomized Controlled Pilot Study
Source: JMIR Res Protoc. 2022 May 17;11(5):e38543. doi: 10.2196/38543 (PMC9157320; doi:10.2196/38543)
Supplement: Multimedia Appendix 1 [file resprot_v11i5e38543_app1.pdf]

SUMMARY STATEMENT
(Privileged Communication)

PROGRAM CONTACT:
Dr. Robert Freeman
301443-8820
rfreeman@mail.nih.gov

Release Date: 11/15/2018
Revised Date:

Application Number: 1 R34 AA026332-01A1

Principal Investigator

LITT, DANA M

Applicant Organization: UNIVERSITY OF NORTH TEXAS HLTH SCI CTR

Review Group: AA-2
Epidemiology, Prevention and Behavior Research Review Subcommittee

Meeting Date: 10/29/2018
Council: JAN 2019
Requested Start: 04/01/2019
RFA/PA: PA18-067
PCC: AP F U

Project Title: Development and Pilot of a Parent Based Intervention for Reducing High-risk Social Networking Site Cognitions, Alcohol Use, and Negative Consequences Among Adolescents
SRG Action: Impact Score:15
Next Steps: Visit https://grants.nih.gov/grants/next_steps.htm
Human Subjects: 30-Human subjects involved - Certified, no SRG concerns
Animal Subjects: 10-No live vertebrate animals involved for competing appl.
Gender: 1A-Both genders, scientifically acceptable
Minority: 1A-Minorities and non-minorities, scientifically acceptable
Children: 1A-Both Children and Adults, scientifically acceptable

Project Year	Direct Costs Requested	Estimated Total Cost
1	150,000	219,000
2	150,000	219,000
3	150,000	219,000
TOTAL	450,000	657,000

ADMINISTRATIVE BUDGET NOTE: The budget shown is the requested budget and has not been adjusted to reflect any recommendations made by reviewers. If an award is planned, the costs will be calculated by Institute grants management staff based on the recommendations outlined below in the COMMITTEE BUDGET RECOMMENDATIONS section.

1R34AA026332-01A1 LITT, DANA

RESUME AND SUMMARY OF DISCUSSION: This is a resubmitted R34 application, which proposes to develop a parent-based intervention to increase parent-teen communication regarding alcohol use and exposure to alcohol-related content on Social Network Sites (SNS). Reviewers found the revised application to be highly responsive to earlier critiques. Two of the substantial improvements that were made in this resubmission were the inclusion of a Treatment as Usual (TAU) condition and moving the format to web-based rather than paper-and-pencil. A few minor and addressable weaknesses were identified. These included differential recruitment and retention procedures for the TAU, if the intervention has a potential to maximally engages the youth and parents, and if parents' have aptitude and comfort for this type communications. In addition to this, concern was raised about cultural differences in parenting as the study population is expected to be comprised of approximately 1/3 Latino families. The panel rated the proposal within Exertional to Outstanding range.

DESCRIPTION (provided by applicant): The prevalence of underage alcohol use continues to be a public health concern. Numerous studies have reported associations between teen drinking tendencies and parental attitudes and beliefs, parental awareness of teen drinking, parental monitoring and the quality of the parent–teen relationship and communication. The extensive work in this area has resulted in parent-based intervention (PBI) efforts to prevent or reduce adolescent alcohol use. Several independent studies have indicated that teens whose parents received a PBI reported less alcohol use and fewer alcohol-related consequences. Despite these strengths, one major limitation of PBI is that they do not currently take into account the large role that social networking sites (SNS) use plays in adolescents' lives and in relation to their alcohol use. Most (90%) adolescents are on SNS, and their Facebook, Instagram, and Twitter profiles include alcohol content. Thus, adolescents are making and exposed to SNS alcohol displays and these displays are associated with high-risk drinking cognitions and alcohol use. As such, we propose to develop and refine an interactive PBI designed to reduce high-risk SNS cognitions (i.e. attitudes and norms), alcohol use, and negative consequences among adolescents. To achieve study aims, we propose an iterative process of focus groups in order to develop and refine the interactive PBI to be delivered in the pilot study with 1 and 6-month follow-up among 100 parent/teen dyads. The objective of this R34 application is to establish feasibility and acceptability of the newly developed interactive PBI that focuses on the role of SNS in adolescent alcohol use as well as to determine preliminary effect sizes for future studies. Determining an efficacious way to reduce alcohol use and high-risk alcohol display cognitions affords future research the opportunity to make use of social network-based interventions, thus the proposed research has great potential to serve as a catalyst for future research.

PUBLIC HEALTH RELEVANCE

The central goal of this application is to develop and pilot an interactive parent-based intervention (PBI) aimed at reducing high-risk social networking site cognitions, alcohol use, and negative consequences among adolescents. This overarching aim will be achieved by conducting two phases of research: a) focus groups for parents and teens and b) pilot study with randomization to an interactive PBI or active control with 1 and 6-month follow-up surveys among 100 parent/teen dyads to determine feasibility, acceptability, and preliminary effect sizes of a PBI (to estimate power and sample sizes for a future R01 application). The proposed study will address each of these goals in turn by enrolling local samples of parents/guardians and their teen children ages 15-20.

CRITIQUE 1

Significance: 1
Investigator(s): 2
Innovation: 2
Approach: 3

Environment: 2

Overall Impact:

This proposal seeks to build on the success of a parent-based intervention for adolescent alcohol use prevention by making it web-based and interactive as well as adding a component to address the problematic influence of alcohol depictions/posting on social network sites. There are a number of factors that contribute to the overall impact of the proposed project including: the potential of developing a prevention tool that would be likely to have a positive effect given previous research, the likely appeal to parents, the current lack of interventions or parent training to address SNS influence, and the readily disenable nature of a web-based intervention. The scientific premise for the study is strong. The design of the project and the research team positively affected the overall impact, although several minor weaknesses are noted to the approach.

1. Significance:

Strengths

- A strong findings-based argument is made for the importance of parents as well as social network site (SNS) use in alcohol outcomes for adolescents, including newer longitudinal research findings providing more of a basis on which to infer causal associations – suggesting that both are important targets for prevention efforts.
- A strong argument is made that parents are in need of training on how to influence drinking and SNS use in effective ways for prevention.
- The interactive web-based format of the PBI with SNS intervention has the potential to be more efficacious than the currently available PBI.
- The focus on SNS has a high potential to appeal to parents given the important role of SNS in youth's lives and lack of general knowledge regarding how to parent with respect to technology.

Weaknesses

- None noted.

2. Investigator(s):

Strengths

- The research team has a record of grant funded research and publications in the areas under investigation, including SNS and internet-based interventions. The expertise of the research team in these areas is established. Each team member is responsible for aspects of the project that are particularly within their area of experience and expertise.
- The team has demonstrated the ability to recruit, retain, coordinate similar studies, and analyze similar data.
- The research team is productive and has successfully worked together in the past.

Weaknesses

- While the research team has considerable experience with the methodologies outlined, both the PI and Co-I are recent transplants to the university where the study will be conducted and to that area of the country. Dallas/Fort Worth has a considerably different demographic than Seattle, which may affect recruitment, focus groups, or the intervention in ways that may not be anticipated. Given that 34% of the sample is anticipated to be Latino there may be cultural differences in parenting practices as well as attitudes toward alcohol that need to be considered. Seeking local consultation or expertise would be worthwhile.

3. Innovation:

Strengths

- Creation of an interactive, web-based intervention with text prompts for PBI is a significant innovation over current handbook-based interventions. This has a higher potential for parent uptake and use than traditional methods of delivery (static, written intervention).
- Including SNS use in an efficacious PBI is a significant innovation that is likely to enhance parental interest and the appeal of the intervention as well as to enhance the effects of PBI.

Weaknesses

- Making static content interactive and web-based is not necessarily paradigm changing.

4. Approach:

Strengths

- A major strength is that the recruitment strategy is broad to reach parents who do and do not currently use SNS.
- Including youths age 18-20 in addition to 15-17-year olds is a strength given continued parental influence for this later age group, as well as the particular need for prevention in this high-risk group for alcohol use disorders.
- The use of focus group feedback from both parents and adolescents from different age ranges to refine the intervention is a major strength. The number of focus groups and large sample sizes are a significant strength.
- The parent and adolescent outcomes measured and longitudinal design, including a six-month follow-up period are a major strength.
- A major strength is that each phase of the project is well designed, including a TAU control that appears to be a good comparison group. Below weaknesses are note; however, each is relatively minor or easily addressed.

Weaknesses

- Although the AUDIT has been used with adolescents, it is a blunt tool for this population. The AUDIT as a measure of alcohol consequences in a general adolescent sample would seem to have quite poor sensitivity (floor effects) given the focus of many of the items on truly problematic drinking. A previous reviewer noted issues with the YAACQ as a measure designed for an older population, the AUDIT is likewise designed for an older sample and to screen for those with misuse in need of intervention. Therefore, it does not seem appropriate for the purposes of a prevention-based intervention and this study. However, its use for assessing risk and as part of the human subject’s protections seems quite appropriate.
- The hypothesis of having differential recruitment, enrollment, and attrition in the intervention vs. TAU is problematic, as this would indicate systematic bias thereby negating the ability to attribute change to the intervention and the benefit of random assignment.
- A minor weakness was that the rationale for the eligibility criteria (as opposed to 13 or 14) was not provided.
- A minor weakness is that the focus group length of 90 minutes seems quite inadequate to cover all of the content planned and elicit feedback. These sessions would need to be considerably longer or use different focus groups to preview different samplings of the content.

- Providing $10 to the PBI group to encourage parents to take their child out to discuss issues seems like it would lower the external validity of the results of the pilot given that this would be an unlikely aspect of the intervention when it is disseminated, as well as raising issues of treating the conditions differently (internal validity).
- There appears that there may be some additional ways in which the intervention groups are treated that may result in differential social desirability, expectancies, and demand characteristics among groups. For example, a letter sent to the PBS parents regarding the goals of the research, but apparently not to the TAU group.
- Ten covariates are listed for inclusion in the GLMM models, this is excessive. I assume that these will be included as appropriate by dependent variable, but this was not stated. It may be worthwhile to examine for potential ethnic differences for inclusion as a covariate or moderator.

5. Environment:

Strengths

- The PIs facilities and resources are well suited to successfully carrying out the proposed study.
- The reputation of The University of North Texas Health Science Center in relation to substance abuse research is not widely known; however, both the PI and the Co-I are seasoned investigators and are at this same institution. This suggests the opportunity for team-based problem solving and collaboration to overcome any potential challenges associated with the new environment.

Weaknesses

- While the facilities section is long, much of what is included is not directly relevant to the current study and factors that may affect the success of the current study are not elaborated on (e.g., dedicated space).

Study Timeline:

Strengths

- Sufficient time is dedicated to each phase of the project.

Weaknesses

- Timeline could include more information to justify the time length devoted to phases of the study.

Protections for Human Subjects:

Acceptable Risks and/or Adequate Protections

- Consent from both parents/guardians and children (minor and adult) appear adequate. Protection of data and identities appears adequate.

Data and Safety Monitoring Plan (Applicable for Clinical Trials Only):

Acceptable

- Protections are in place for participants with dangerous levels of alcohol consumption. Adequate plans are in place to protect participant identities and personal information.

Inclusion of Women, Minorities and Children:

- Sex/Gender: Distribution justified scientifically
- Race/Ethnicity: Distribution justified scientifically
- For NIH-Defined Phase III trials, Plans for valid design and analysis: Not applicable
- Inclusion/Exclusion of Children under 18: Including ages <18; justified scientifically
- Sex/gender, race/ethnicity, and age inclusionary criteria are represented and justified. The study is restricted to adolescents and very young adults age 15-20 and their parents or guardians. This is justified given the parent-based intervention being studied.

Resource Sharing Plans:

- Acceptable

Authentication of Key Biological and/or Chemical Resources:

- Not Applicable (No Relevant Resources)

Budget and Period of Support:

Budget Modifications Recommended (in amount/time)

Recommended budget modifications or possible overlap identified:

- Other than personal no budget justification or details are provided thus it is difficult to judge whether the budget should be recommended without modifications.

CRITIQUE 2

Significance: 1
Investigator(s): 1
Innovation: 2
Approach: 1
Environment: 1

Overall Impact:

This is a revision of an earlier proposal to modify an existing parent-based intervention approach. It seeks to provide parents with information about current teen issues with online social media access, social media use, and alcohol presence on social media. Its goal is to assist parents to more effectively engage in parental mediation of a teen social environment that is deeply saturated with social media sites. Most teens report going online daily, and much of this time is spent on social network sites where one quarter of adolescent profiles include alcohol-related content. Parents often have limited knowledge regarding their children's social media profiles or the types of online experiences they are having. This proposal is for an intervention to bridge this gap in parent knowledge. The intervention seeks to assist parents in a role as socialization agents to counter negative social media influence on drinking and focuses on risky alcohol cognitions in youth. The revision is now clear on ways social media influences are potentially stronger than other areas of media influence in adolescent drinking through their interactive nature and through a diminished separation of online and offline lives, as well as ways in which parent mediation of social media needs to change in order to be effective. The proposal addresses the issue of findings of mixed effects of the existing parent-based intervention and introduces a new component to parent-based intervention that may heighten its impact. The investigator team and environment are strong, and the focus on social media for a parent-based

intervention is innovative. In the revised proposal, instead of consisting entirely of parent readings in paper-based manuals, the parent educational materials are now web based and interactive. However, opportunities to provide parents experiential exposure to the types of immersive experiences of influence their own children are exposed to through alcohol related social media content does not yet seem maximally realized through what is described in the approach. Specific recruitment efforts are now detailed that will be used to facilitate diverse recruitment. The revision now proposes to use as a main alcohol outcome measure a measure that has been validated with adolescents, and outcomes are now extended out to 6 months posttreatment. The GLM model is now precisely described and possible covariates to be explored are now clearly and extensively delineated.

1. Significance:

Strengths

- Parental influences to discourage drinking are strongly associated with reductions in adolescent drinking.
- Teens report being online daily with a majority of time on social network sites; data indicates up to 1⁄4 of adolescent social network site profiles include alcohol-related content; at the same time parents often have limited knowledge regarding this content and youth internet experience in general.
- Parent based intervention approaches have not kept up with social media and the role of social media in adolescent experience and socialization to alcohol.
- There is a need for intervention to bridge this gap by providing parents with information about teen social media use and social media alcohol exposures to assist parental mediation as socialization agent to prevent teen alcohol misuse.
- The revision presents important research findings that, "*There is a robust relationship between exposure to SNS alcohol content and alcohol consumption six months later, which persists even after close friends' drinking was accounted for (Boyle et al., 2016)"* that addresses a question about the significance of this study: does exposure to alcohol displays through social media sites lead to risky alcohol cognitions in ways that are additive to the impacts of more direct life experience in family and peer settings? Further, the authors note: "*adolescents use SNS to reconstruct negative and risky drinking practices into positive outcomes (Hebden et al., 2015; Lyons et al., 2015) in order to avoid acknowledging any implications of or reference to negative consequences associated with drinking (Niland et al., 2014)"* suggesting social media sites may lead to risky alcohol cognitions in ways that are potentially different and more powerful.
- The investigators are now much clearer in their theory and rationale regarding the significance of social media sites in their influence on youth. The revision now describes literature on how traditional media channels cultivated passive observers who were not able to influence content, while social media sites allow users to participate in how content is created and shared. The revision also notes how parents often find it difficult to relate to how for their digitally savvy children online lives are an extension of their offline lives, and importantly, given social media sites' interactivity, immersive virtual environments, and mediated communication, general parental mediation strategies for more traditional forms of media may not be effective for this new medium.
- Given this universal intervention can be offered with ease of dissemination and at low-cost, its modest intervention effects, on a population level, can be significant.
- Previous research on parent-based intervention has produced inconsistent findings and modest effect sizes; however, this approach may enhance its effects in moving from static formats of parent handbooks to web-based, more interactive formats.

Weaknesses

- None noted

2. Investigator(s):

Strengths

- Dr. Litt has strong history of work with parent-based intervention, clinical trials research, and recently completed research on impact of alcohol displays on social networking sites on adolescent alcohol use.
- Dr. Lewis has a long history of success conducting theory-driven research focused on the role of social influence on the etiology and prevention of drinking along with work on 10 NIAAA- and NIDA-funded longitudinal research projects.
- As consultants, Dr. Turrisi has background to assist in development and refinement of the parent intervention while Dr. Moreno can provide focus group questions, refining of intervention materials, measurement development, and design.

Weaknesses

- None noted

3. Innovation:

Strengths

- The intervention would train and assist parents to address social network site social influence.
- The intervention inserts education on social network sites and their potential influence on risky alcohol cognitions into an established evidence-based parent-based intervention approach.
- A strength in this revised proposal is, in keeping with the project focus upon digital media's impacts upon youth and a migration of sources of social influence on youth to social media, the proposed intervention materials are also moving from paper to web-based, interactive, and accompanied by text message prompts.

Weaknesses

- There remains an unrealized opportunity for the web-based interactive training materials to actually provide experiential examples of the interactivity, immersive virtual environments, and mediated communication that would provide adults with the kinds of experiences their children are currently encountering; this would enhance parent understanding of these digital sources of influence and the vanishing distinction between online and offline lives.

4. Approach:

Strengths

- Excellent focus group and cognitive interviewing strategies in intervention development efforts.
- An addition to the revised design is to now measure parental education/literacy as a covariate.
- A 6 month follow up assessment provides additional evidence especially with regards to the sustainability of impact over time.
- The revised proposal now emphasizes feasibility and acceptability of the intervention as critical research questions for the pilot implementation study, and precisely operationalizes each.

- The proposed analysis to explore treatment differences and determine preliminary effect sizes is now thoroughly documented. The GLMM is now presented in the revision through conventional mixed model conventional notation , and in addition, covariates to be considered are presented, and include age, gender, parent-teen relationship satisfaction, adult attitudes and teen perceptions of their parents' attitudes toward teen alcohol use and alcohol displays on SNS, parental monitoring, parental education, baseline levels of teen drinking and consequences, SNS use, and baseline levels of communication about alcohol use and SNS will be used as covariates.
- Retention efforts are significant and are now extensively described in the revision, and include efforts directed towards enhanced diversity in the sample.
- Instead of administering to adolescents the Young Adult Alcohol Consequences Questionnaire, in the revised proposal, both parents and youth will now complete the Alcohol Use Disorders Identification Test (α = .85; Babor et al., 2001) which has been validated in both adult and adolescent samples.
- Outcomes in the revised proposal now extend to 6 months instead of one month, providing a test of durability of intervention effects.

Weaknesses

- None noted

5. Environment:

Strengths

- University of North Texas Health Science Center School of Public Health includes a Department of Biostatistics and Epidemiology which houses the Biostatistics and Epidemiology Consulting and Collaboration Services and University of North Texas Health Science Center Division of Research and Innovation is a center for research assistance including compliance and regulatory assistance
- Excellent capabilities in software development as well as analytical support.

Weaknesses

- Elements related to clinical trials support appropriate for the trial proposed that facilitate investigator ability to conduct the trial and meet its regulatory and reporting requirements are not specified.

Study Timeline:

Strengths

- The timeline describes time periods for basic elements of the study including start-up activities.

Weaknesses

- The timeline does not take into account anticipated rate of enrollment and delineate the planned follow-up assessments.
- The timeline does not describe how the project incorporates efficiencies and utilizes existing resources at the home institutions.

- Potential challenges and corresponding solutions are not discussed, such as strategies that can be implemented in the event of enrollment shortfalls

Protections for Human Subjects:

Acceptable Risks and/or Adequate Protections

- The protections of human subjects are well written, thorough and extensive in its considerations of potential risks, ways to mitigate risks, and benefits to participants.

Data and Safety Monitoring Plan (Applicable for Clinical Trials Only):

Acceptable

- The data safety management plan is thorough and well-conceived. A plan is now in place for identifying and referring individuals who report significantly worsening alcohol use trajectories as well as consumption of potentially lethal doses of alcohol (BAC's above .35) and potential for alcohol use disorder as measured on the AUDIT. Once identified, these individuals will be contacted by the PI within 48 hours to explain their risk and to provide referral. The investigators note in their response ot reviews "all teens" will receive information about alcohol use regardless of their personal risk to address confidentiality concerns. The investigators need to clarify what is meant by "teens" and if this means under 18 or all youth under 21, for which drinking is illegal. The investigators clarify that parents will not be notified specifically about their children's level of risk but will receive general information instead. The investigators in this revision have clarified that while ultimate outcomes from the intervention impact youth and young adults, including children from age 15-17, as reported through self-report assessments, the intervention procedures are entirely focused on parents. Therefore, though the study involves children, which are a vulnerable population in the NIH definition, the intervention activities occur with their parents, and is therefore seems best understood as a Phase II clinical trial that does not involve participants from a vulnerable population, and a DSMB is not required.

Inclusion of Women, Minorities and Children:

- Sex/Gender: Distribution justified scientifically
- Race/Ethnicity: Distribution justified scientifically
- For NIH-Defined Phase III trials, Plans for valid design and analysis: Not applicable
- Inclusion/Exclusion of Children under 18: Including ages <18; justified scientifically
- Inclusion of children, and gender and ethnicity distributions are well justified.

Applications from Foreign Organizations:

- Not Applicable (No Foreign Organizations)

Select Agents:

- Not Applicable (No Select Agents)

Resource Sharing Plans:

- Acceptable

Authentication of Key Biological and/or Chemical Resources:

- Not Applicable (No Relevant Resources)

Budget and Period of Support:

- Recommend as Requested

CRITIQUE 3

Significance: 1
Investigator(s): 1
Innovation: 1
Approach: 2
Environment: 1

Overall Impact:

This revised R34 application, describes a plan to develop a parent-based intervention to increase parent-teen communication regarding alcohol use and exposure to alcohol-related content on Social Network Sites (SNS). Strengths of the initial application, including a novel idea of considerable potential value for the field, a strong research team, and a solid approach all remain in this revised application. Further, investigators have been highly responsive to reviewer critiques, substantially modifying their approach to be more current (online rather than mailed handbook) and more developmentally sensitive. Many details have been considered and planned for, and the plan for development and testing of this intervention is strong. Enthusiasm is high for this excellent application.

1. Significance:

Strengths

- SNS are a big part of the daily lives of adolescents, and have the potential to influence adolescent drinking. Existing parent-based interventions do not address this form of social influence and as such, the proposed intervention could address a gap in the battery of available prevention options in an important way.
- The intervention is designed to be applied to multiple types of SNS, and thus will have broad application.

Weaknesses

- None noted.

2. Investigator(s):

Strengths

- This is a strong investigative team with relevant experience and expertise to successfully execute the proposed project.
- Dr. Litt is a productive investigator who has a strong track record in the area of adolescent and young adult alcohol misuse. She also has done much work in the areas of social influences – including online influences – on alcohol risk.
- The contributions of each team member are appropriate for the project and well-justified in the application.

- Dr. Turrisi has a well-established record of expertise in parent-based intervention for reducing alcohol misuse.

Weaknesses

- None noted.

3. Innovation:

Strengths

- Existing parent-based interventions to reduce adolescent and young adult drinking do not address the role of SNS. For this reason, the proposed intervention is innovative.

Weaknesses

- None noted.

4. Approach:

Strengths

- Investigators have made a number of changes to their approach, all of which render the proposed intervention more appropriate to the population and problem on which they seek to intervene. This includes the switch from a mailed handbook to a web-based intervention with text prompts. This seems much more accessible and current and is a substantial improvement over earlier proposed procedure. Also, the plan to develop the intervention with different age/developmental stage in mind is an excellent shift. Finally, the authors now plan to gather more and more detailed information about feasibility and acceptability in the developmental stages of the project.
- Similarly, changes to the Significance section of the application make a stronger and more compelling case for the role of SNS is drinking risk.

Weaknesses

- There still are concerns about how well-prepared parents are to have targeted and productive conversations with their adolescents about alcohol use and social media. It would seem valuable to add some measures of this aspect of feasibility. These might include parents' comfort level with this material, sense of expertise or mastery, and the parents' own social media use.
- It also would seem useful to get some kind of measure of parent-child relationship.

5. Environment:

Strengths

- This is an outstanding environment in which to conduct the proposed work.

Weaknesses

- It would be valuable for investigators to consider recruitment and retention issues that may arise in what is a new environment for some of these investigators, specifically with respect to ethnic minority participation.

Study Timeline:

Strengths

- The study timeline is appropriate for the scale and time constraints of the proposed work.

Weaknesses

- None noted.

Protections for Human Subjects:

Acceptable Risks and/or Adequate Protections

Data and Safety Monitoring Plan (Applicable for Clinical Trials Only):

Acceptable

Inclusion of Women, Minorities and Children:

- Sex/Gender: Distribution justified scientifically
- Race/Ethnicity: Distribution justified scientifically
- For NIH-Defined Phase III trials, Plans for valid design and analysis:
- Inclusion/Exclusion of Children under 18: Including ages <18; justified scientifically

Resubmission:

- The investigators have been highly responsive to earlier critiques. Introduction to the revised application and accompanying changes to the application were thoughtful and thorough.

Resource Sharing Plans:

- Acceptable

Budget and Period of Support:

- Recommend as Requested

THE FOLLOWING SECTIONS WERE PREPARED BY THE SCIENTIFIC REVIEW OFFICER TO SUMMARIZE THE OUTCOME OF DISCUSSIONS OF THE REVIEW COMMITTEE, OR REVIEWERS' WRITTEN CRITIQUES, ON THE FOLLOWING ISSUES:

PROTECTION OF HUMAN SUBJECTS: ACCEPTABLE

INCLUSION OF WOMEN PLAN: ACCEPTABLE

INCLUSION OF MINORITIES PLAN: ACCEPTABLE

INCLUSION OF CHILDREN PLAN: ACCEPTABLE

COMMITTEE BUDGET RECOMMENDATIONS: The budget was recommended as requested.

Footnotes for 1 R34 AA026332-01A1; PI Name: LITT, DANA M

NIH has modified its policy regarding the receipt of resubmissions (amended applications). See Guide Notice NOT-OD-14-074 at http://grants.nih.gov/grants/guide/notice-files/NOT-OD-14-074.html. The impact/priority score is calculated after discussion of an application by averaging the overall scores (1-9) given by all voting reviewers on the committee and multiplying by 10. The criterion scores are submitted prior to the meeting by the individual reviewers assigned to an application, and are not discussed specifically at the review meeting or calculated into the overall impact score. Some applications also receive a percentile ranking. For details on the review process, see http://grants.nih.gov/grants/peer_review_process.htm#scoring.

MEETING ROSTER

Epidemiology, Prevention and Behavior Research Review Subcommittee
National Institute on Alcohol Abuse and Alcoholism Initial Review Group
NATIONAL INSTITUTE ON ALCOHOL ABUSE AND ALCOHOLISM
AA-2
10/29/2018

Notice of NIH Policy to All Applicants: Meeting rosters are provided for information purposes only. Applicant investigators and institutional officials must not communicate directly with study section members about an application before or after the review. Failure to observe this policy will create a serious breach of integrity in the peer review process, and may lead to actions outlined in NOT-OD-14-073 at https://grants.nih.gov/grants/guide/notice-files/NOT-OD-14-073.html and NOT-OD-15-106 at https://grants.nih.gov/grants/guide/notice-files/NOT-OD-15-106.html, including removal of the application from immediate review.

CHAIRPERSON(S)

NEIGHBORS, CLAYTON, PHD
PROFESSOR AND DIRECTOR
DEPARTMENT OF PSYCHOLOGY
UNIVERSITY OF HOUSTON
HOUSTON, TX 77204

MEMBERS

ALLEN, JAMES R., PHD
PROFESSOR
DEPARTMENT OF BIOBEHAVIORAL HEALTH & POPULATION SCIENCES
UNIVERSITY OF MINNESOTA MEDICAL SCHOOL, DULUTH
DULUTH , MN 55812

CAETANO, RAUL, MD, PHD
SENIOR RESEARCH SCIENTIST
PREVENTION RESEARCH CENTER
PACIFIC INSTITUTE FOR RESEARCH AND EVALUATION
OAKLAND, CA 94612

DERMODY, SARAH SIODMOK, PHD *

FELL, JAMES C.
PRINCIPAL RESEARCH SCIENTIST
DEPARTMENT OF ECONOMICS, JUSTICE, AND SOCIETY
NATIONAL OPINION RESEARCH CENTER AT THE UNIVERSITY OF CHICAGO
BETHESDA, MD 20814

FURR-HOLDEN, C. DEBRA, PHD
C.S. MOTT ENDOWED PROFESSOR OF PUBLIC HEALTH
PROFESSOR
DEPARTMENT OF EPIDEMIOLOGY AND BIOSTATISTICS
COLLEGE OF HUMAN MEDICINE
MICHIGAN STATE UNIVERSITY
FLINT, MI 48502

GIZER, IAN ROBERT, PHD
ASSOCIATE PROFESSOR
DEPARTMENT OF PSYCHOLOGICAL SCIENCES
UNIVERSITY OF MISSOURI, COLUMBIA
COLUMBIA, MO 65211

GONZALEZ, VIVIAN M., PHD *
PSYCHOLOGY DEPARTMENT
UNIVERSITY OF ALASKA ANCHORAGE
ANCHORAGE, AK 99508

LABRIE, JOSEPH W, PHD *
PROFESSOR
DEPARTMENT OF PSYCHOLOGY
LOYOLA MARYMOUNT UNIVERSITY
LOS ANGELES, CA 90045-2659

MCGUE, MATTHEW K., PHD
PROFESSOR
DEPARTMENT OF PSYCHOLOGY
MEMBER, INSTITUTE OF HUMAN GENETICS
UNIVERSITY OF MINNESOTA
MINNEAPOLIS, MN 55455

ONDERSMA, STEVEN J, PHD *
PROFESSOR
MERRILL PALMER SKILLMAN INSTITUTE
SCHOOL OF MEDICINE
WAYNE STATE UNIVERSITY
DETROIT, MI 48236

PATRICK, MEGAN ELIZABETH, PHD
RESEARCH ASSOCIATE PROFESSOR
SURVEY RESEARCH CENTER
INSTITUTE FOR SOCIAL RESEARCH
UNIVERSITY OF MICHIGAN
ANN ARBOR, MI 48106-1248

READ, JENNIFER P., PHD
PROFESSOR
DEPARTMENT OF PSYCHOLOGY
UNIVERSITY AT BUFFALO
THE STATE UNIVERSITY OF NEW YORK
BUFFALO, NY 14260

TONIGAN, J SCOTT, PHD *
PROFESSOR
DEPARTMENT OF PSYCHOLOGY
UNIVERSITY OF NEW MEXICO
ALBUQUERQUE, NM 87106

SCIENTIFIC REVIEW OFFICER

GHAMBARYAN, ANNA, MD, PHD
SCIENTIFIC REVIEW OFFICER
EXTRAMURAL PROJECT REVIEW BRANCH
OFFICE OF EXTRAMURAL ACTIVITIES
NATIONAL INSTITUTE ON ALCOHOL ABUSE AND ALCOHOLISM
NATIONAL INSTITUTES OF HEALTH
ROCKVILLE, MD 20852

EXTRAMURAL SUPPORT ASSISTANT

FULTON, THELMA
EXTRAMURAL SUPPORT ASSISTANT
OFFICE OF EXTRAMURAL ACTIVITIES
NATIONAL INSTITUTE ON ALCOHOL ABUSE AND ALCOHOLISM
NATIONAL INSTITUTES OF HEALTH
ROCKVILLE, MD 20892-9304

* Temporary Member. For grant applications, temporary members may participate in the entire meeting or may review only selected applications as needed.

Consultants are required to absent themselves from the room during the review of any application if their presence would constitute or appear to constitute a conflict of interest.
